# Inadequate Dietary Phosphorus Levels Cause Skeletal Anomalies and Alter Osteocalcin Gene Expression in Zebrafish

**DOI:** 10.3390/ijms19020364

**Published:** 2018-01-25

**Authors:** Juliana M. Costa, Maria M. P. Sartori, Nivaldo F. do Nascimento, Samir M. Kadri, Paulo E. M. Ribolla, Danillo Pinhal, Luiz E. Pezzato

**Affiliations:** 1 Department of Genetics, Institute of Biosciences, Sao Paulo State University (UNESP), Botucatu, Sao Paulo 18618-970, Brazil; 2 Department of Crop Science, College of Agricultural Sciences, Sao Paulo State University, Botucatu, Sao Paulo 18610-307, Brazil; mmpsartori@fca.unesp.br; 3 Aquaculture Center, Sao Paulo State University (CAUNESP), Jaboticabal, Sao Paulo 14884-900, Brazil; nivaldotec@yahoo.com.br; 4 College of Veterinary and Animal Science, Sao Paulo State University (UNESP), Botucatu, Sao Paulo 18618-970, Brazil; samirkbr@yahoo.com.br (S.M.K.); epezzato@fmvz.unesp.br (L.E.P.); 5 Botucatu Biotechnology Institute, Sao Paulo State University (UNESP), Botucatu, Sao Paulo 18618-970, Brazil; pribolla@ibb.unesp.br

**Keywords:** osteocalcin, bone mineralization, abnormalities

## Abstract

Phosphorus (P) is an essential mineral for the development and maintenance of the vertebrate skeletal system. Modulation of P levels is believed to influence metabolism and the physiological responses of gene expression. In this study, we investigated the influence of dietary P on skeletal deformities and osteocalcin gene expression in zebrafish (*Danio rerio*), and sought to determine appropriate levels in a diet. We analyzed a total of 450 zebrafish within 31 days of hatching. Animals were distributed in a completely randomized experimental design that consisted of five replications. After an eight-week experiment, fish were diaphanized to evaluate cranial and spinal bone deformities. Increases in dietary phosphorus were inversely proportional to the occurrence of partial spine fusions, the absence of spine fusions, absence of parallelism between spines, intervertebral spacing, vertebral compression, scoliosis, lordosis, ankylosis, fin caudal insertion, and craniofacial deformities. Additionally, osteocalcin expression was inversely correlated to P levels, suggesting a physiological recovery response for bone mineralization deficiency. Our data showed that dietary P concentration was a critical factor in the occurrence of zebrafish skeletal abnormalities. We concluded that 1.55% P in the diet significantly reduces the appearance of skeletal deformities and favors adequate bone mineralization through the adjustment of osteocalcin expression.

## 1. Introduction

Phosphorus (P) is an essential macromineral and is one of the major components of the inorganic portion of fish diets, along with calcium [1]. This mineral acts on coenzymes, phospholipids, nucleic acids, and energy transfer through the breakdown of adenosine triphosphate (ATP) for metabolic processes and muscle contraction [2]. P is required in the formation of hard tissue structures, such as bones, teeth, and scales [3], and is also necessary for soft tissue, where it works as a component of genetic material and as a structural constituent of cell membranes and intracellular organelles [4]. 

Bone tissue is a reservoir of phosphorus, calcium, and other ions, which are made available in blood and cellular fluids [2,3,4]. The main bone structure in teleost fishes is the backbone, which is also called the axial skeleton [5]. This postcranial endoskeleton is segmented into vertebrae, which carry neural arches extended by neural spines (dorsal) and hemal arches extended by hemal spines (ventral) [6]. Many studies have investigated the associations of diets with skeletal deformities in teleost body segments, including the cranial, abdominal, and the caudal regions [7,8,9]. Currently, skeletal deformities in fish are a subject of growing interest, particularly in relation to farmed fish, due to economic losses and skeletal diseases in model fish for biomedical research [10]. Ferreri et al. [11] compared wild and cultivated zebrafish and found caudal region deformities in 86% and 100% of the animals, respectively.

At the molecular level, bone mineralization is regulated by proteins that are synthesized by osteoblasts. Osteocalcin (OC), or the bone Gla protein (BGP), is considered a mature bone tissue marker because it is only expressed in differentiated osteoblasts [12]. This protein is important to body’s metabolism, as it modulates bone mineralization [13] and regulates bone resorption [14]. Osteocalcin also acts as a hormone in the body, mediating the crosstalk between bone and other tissues and organs [15], with a recognized impact on fertility [16], exercise capability [17,18], energy metabolism, and fatty acid breakdown [19].

In fish, the vertebral bodies and spines show high OC concentrations, as demineralization is more intensive in these skeletal elements, whereas the operculum and dermal scales show low concentrations [20]. This variable OC concentration can be caused by a decrease in osteoblast maturation and augmented bone resorption [14]. OC contributes to the formation of bone mineral hydroxylapatite, which contains phosphorus in the form of inorganic phosphate [21]. This implies that phosphorus levels may impact or correlate with OC expression. 

Currently, one of the most widely used model organisms in toxicology, neurobiology, and molecular genetics studies is the zebrafish (*Danio rerio*), a freshwater teleost. Some of its remarkable qualities are its rapid development [22], high reproductive capacity [23], and transparent larval development [24], and there is a comprehensive range of molecular tools and genomic data available for use [25]. This species has also been considered as an excellent model for fish growth and nutrition assessment [26], as well as for the study of regeneration and skeletal deformities using in vivo imaging techniques [27].

Despite the reputation of zebrafish in science, there are only a few studies on their nutritional requirements [26,28]. When confined in research laboratories, the nutritional requirements of zebrafish must be met via feeding, and diets must be balanced in order to supply the minerals required to maintain an optimal metabolism [29].

Proper vivarium animal nutrition is important, given that deficient diets can yield unwanted experimental biases. Thus, this study evaluated the potential influence of P levels on skeletal deformities, and on the relative expression of the OC protein-encoding gene; the most appropriate dietary phosphorus level for zebrafish was also determined. Skeletal anomalies and gene expression levels were determined by morphological inspection of diaphanized fish and by qRT-PCR experiments, respectively. 

## 2. Results

**Figure 1:**
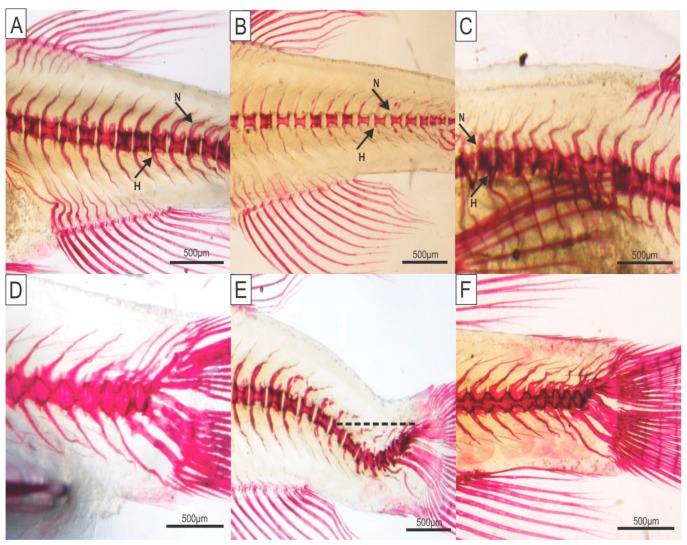
Deficient dietary phosphorus intake impairs zebrafish (*Danio rerio*) skeletal development. Vertebral and spinal anomalies are shown in zebrafish fed with T1–T6 levels of phosphorus in the diet (T1 = 0.35%; T2 = 0.65%; T3 = 0.95%; T4 = 1.25%; T5 = 1.55%; T6 = 1.85%). (**A**) Partial fusion in neural and hemal spines in T2 fishes; (**B**) Absence of neural and hemal spine fusions in T1 fishes; (**C**) Absence of parallelism between neural and hemal spines in T2 fishes; (**D**–**F**) Caudal vertebral and spinal anomalies; (**D**) Normal vertebral column in T5 fishes; (**E**) Deformed vertebral column at the caudal fin region (the dotted line represents the estimated normality) in T1 fishes; and (**F**) anchylosis in T1 fishes. N and H arrows point to the neural and hemal spines, respectively. Scale bar: 500 μm.

A large number of skeletal deformities were detected in zebrafish (Table 1) in response to different levels of dietary P in six treatments (T1–T6; T1 = 0.35%; T2 = 0.65%; T3 = 0.95%; T4 = 1.25%; T5 = 1.55%; T6 = 1.85%). Partial neural and hemal spine fusions (Figure 1) were observed in T1, T2, T3, and T4 animals, but were absent in T5 and T6 animals (*p* ≤ 0.0001). The reduction in spine fusion was proportional to the increase in dietary P levels, and T6 fish did not display bone deformities (Table 1). The absence of parallelism between neural and hemal spines was critical in T1 fish, while deformities were not observed in T6 fish (*p* ≤ 0.0001).

Changes in intervertebral spacing were not observed in T4, T5, and T6 fish (*p* ≤ 0.0001). Higher vertebral spacing occurred in T1 fish, corresponding to 80% of the total vertebrae. There were more T2 and T3 individuals with compressed vertebrae (*p* ≤ 0.0001), whereas T5 and T6 fish did not show such deformities. 

**Figure 2:**
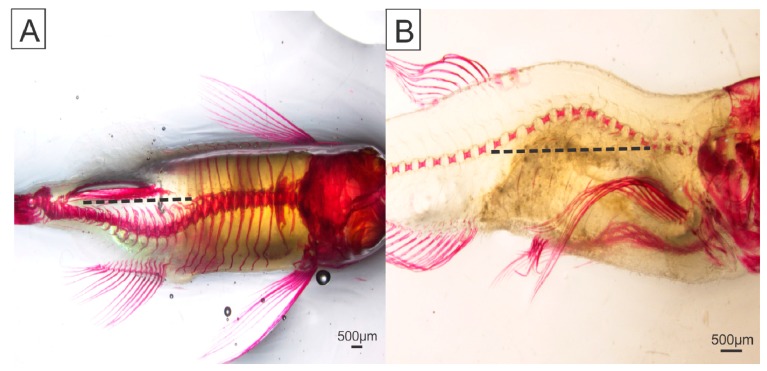
Skeletal anomalies of zebrafish (*Danio rerio*) fed with low dietary phosphorus levels. (**A**) Lordosis and (**B**) scoliosis anomalies in T1 fish. (**A**,**B**) The dotted lines represent the estimated normal vertebral column curvatures. Scale bar: 500 μm.

**Figure 3:**
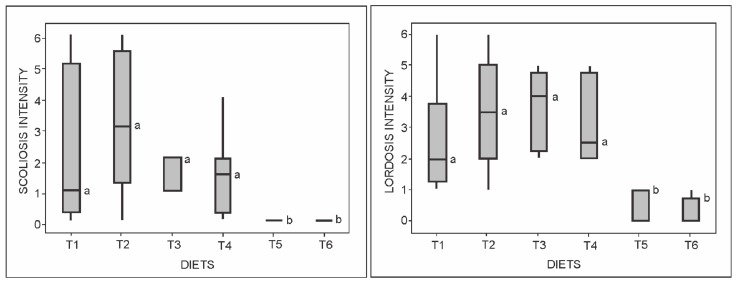
Low-level box plots depicting the intensities of scoliosis and lordosis anomalies in zebrafish (*Danio rerio*) fed with variable (T1–T6) dietary phosphorus levels. Whiskers represent the 95% confidence interval of the data set. ^a,b^ Medians labeled with distinct superscript letters are significantly different (Kruskal-Wallis test, *p* < 0.0001).

**Figure 4:**
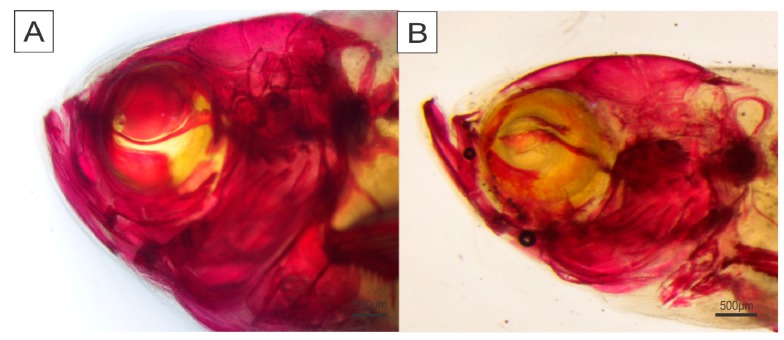
Craniofacial anomaly in zebrafish (*Danio rerio*) fed diets with different phosphorus levels (T1–T6; T1 = 0.35%; T2 = 0.65%; T3 = 0.95%; T4 = 1.25%; T5 = 1.55%; T6 = 1.85%). (**A**) Normal head (T5 fish); (**B**) lower mandible larger than upper mandible (undershot) (T1 fish). Scale bar: 500 μm.

We also determined the severity of skeletal abnormalities after the trial period in the diaphanized fish (parameters are detailed in Materials and Methods). The intensities of scoliosis and lordosis deformities (Figure 2) were severe in T1, T2, T3, and T4 animals (*p* ≤ 0.0001), with scores ranging from 1 to 3 for scoliosis and 2 to 4 for lordosis (Figure 3). T5 and T6 animals showed lower scoliosis and lordosis intensities, with no significant differences between them (*p* ≤ 0.0001). Severe craniofacial deformities were also observed in T1, T2, T3, and T4 animals, and significantly differed from T5 and T6 fish, which did not exhibit any morphological alterations (Figure 4).

**Figure 5:**
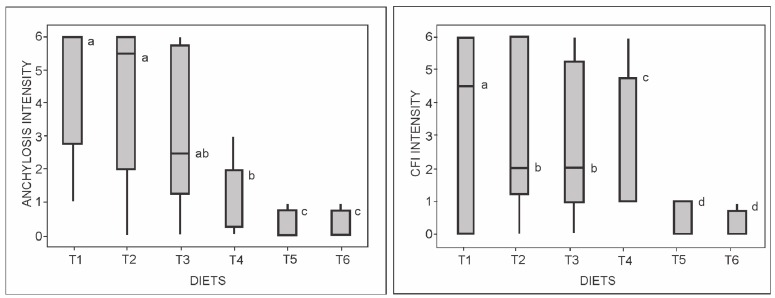
Anchylosis and caudal fin (CFI) anomaly boxplots of zebrafish (*Danio rerio*) fed diets with different phosphorus levels (T1–T6). ^a,b,c,d^ Medians with different superscript letters are significantly different (Kruskal-Wallis test, anchylosis (*p* ≤ 0.0001) and CFI (*p* ≤ 0.003).

**Figure 6:**
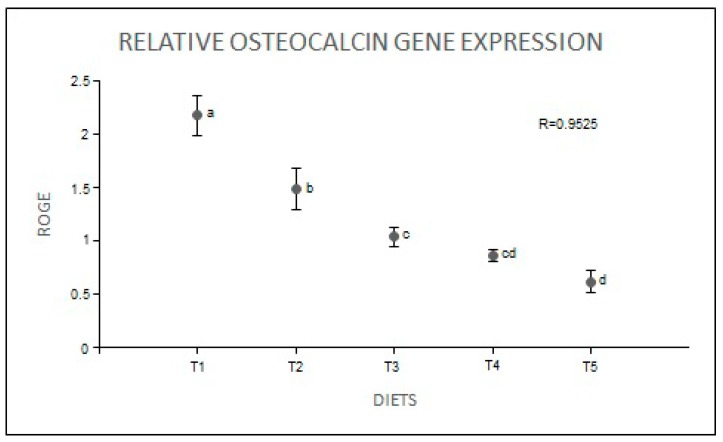
Relative osteocalcin (OC) gene expression (ROGE) of zebrafish (*Danio rerio*) fed with various dietary phosphorus levels (T1–T5). ^a,b,c,d^ Superscript letters denote significant differences (Tukey’s multiple-range test, *p* < 0.05).

The intensities of ankylosis and caudal fin (CFI) anomalies were inversely proportional to the increase in dietary P. The intensity of ankylosis was higher in T1 and T2, while the intensity of CFI was higher in T1 animals (Figure 5). The relative expression of the OC protein-encoding gene was negatively correlated to dietary P levels (Figure 6).

## 3. Discussion

Several other studies also found that skeletal deformities [30,31] or changes in bone mineralization [9] were the main morphological signs found in fish fed P-deficient diets. Bone malformation has been closely linked to fish nutrition, as phosphorus affects skeletal development [32,33]. Thus, low bone mineralization, caused by either mineral deposition failure in bone or bone resorption, is responsible for the high incidence of bone deformities in fish fed P-deficient diets [34]. Notably, OC was previously shown to interfere with bone mineralization and remodeling [20], which matches the expression patterns found in this study.

Due to the roles they play in body support and movement, fish skeletons are exposed to strong mechanical stress during swimming. In particular, the caudal and caudal fin regions of the zebrafish vertebral column are directly linked to the skeleton and the act of swimming [35]. These bone regions are mostly affected by dietary P concentrations, since they influence the direction and intensity of swimming pulses. This functional role might also explain the high degree of abnormalities in these regions, as they would require greater P concentrations for mineralization and mineral replacement due to bone wear. Notwithstanding, additional experimental evidence supporting this attractive hypothesis is still required.

The highest incidence of vertebral anomalies in zebrafish fed with the T1, T2, and T3 diets (with insufficient P) confirmed the influence of P in low bone mineralization. Despite T1 and T2 animals having the largest number of neural and/or hemal spines with partial fusions (some of the most severe deformities affecting the vertebral body [7,36,37,38]), we supposed that such deformities did not cause any external damage to the animals, as the muscles involved with this bone structure assist in not causing damage in swimming. The combination of high intervertebral spacing and vertebral compression displayed by animals fed with lower dietary P levels could be related to the overload imposed on the zebrafish spinal column by the biological needs of fish growth [7]. Without sufficient P for the proper development of the spinal column, an organism might space its vertebrae as a compensatory mechanism to allow constant growth. We envision that high intervertebral spacing reduces the mechanical support of the swim bladder; therefore, as a compensatory action, the fish uses its fins excessively. This combination causes abnormalities in the axis column, such as lordosis and scoliosis, and might help to explain the instances of severe lordosis and scoliosis observed in zebrafish under the T1, T2, T3, and T4 diet regimens.

Similar to our study, P-deficiency signs were observed in the vertebrae of haddock (*Melanogrammus aeglefinus*) [38] and silver perch (*Bidyanus bidyanus*) [39], reinforcing that spinal column is mostly affected by low mineral intake.

Deformities in the caudal region, ankylosis, and SE can occur as vertebrae in this region are under constant injury, and also because vertebrae are larger and more mineralized for mechanical resistance [10,39]. Furthermore, the musculature in this region is responsible for the driving force during swimming. 

Craniofacial deformities (Figure 6) were observed more frequently in animals fed with P-deficient diets (T1, T2, T3, and T4). Consequently, fish can display a significant decrease in food intake, which reduces body growth and weight, thus leading to a loss in commercial value [40]. These issues increase fish farm costs and can bias research results.

Nutrient concentrations in a diet can affect the metabolism and physiological responses of gene expression [41]. Thus, the level of dietary P can directly affect skeletal development by influencing the specific expression of genes, such as OC. This was clearly observed by Shih et al. [42] while studying bone tissue formation by stem cells. These authors demonstrated the role of phosphate in the osteogenic involvement of stem cells, and their relationship to relative OC expression, which was modulated by bone mineralization. Studies focusing on OC regulatory mechanisms have shown that this gene produces a bone-modulatory protein [43,44,45] that can negatively or positively interfere with bone deposition under variable physiological contexts. More specifically, the two-fold function refers to OC regulation over bone remodeling by controlling osteoblast/osteoclast activity, and also by managing bone mineralization (reviewed in [17]).

Considering a scenario of negative OC influence, a higher expression of osteocalcin in animals fed with P-deficient diets (T1 and T2) could be a physiological recovery response to bone mineralization deficiency, given that P must be made available in cells to supply basic metabolic requirements (e.g., as a part of key constitutive molecules, such as ATP and nucleotides). This causes low deposits of P in bone tissues. Supporting this idea, low OC expression was inversely correlated to an increase in bone mass and the functional quality of bone tissue in mice [43,44,45], implying that OC could negatively modulate bone formation by, most likely, being involved in the inhibition of osteoblastic activity [17]. Conversely, in animals fed deficient dietary P, high OC expression could have a positive effect on bone mineralization by triggering a compensatory response that increases the recruitment and maturation of hydroxylapatite as a physiological attempt to maintain bone integrity [44]. In addition, it is known that OC acts as an inhibitor of phosphate, regulating endopeptidase homolog X-linked (PHEX), a mechanism of bone metabolism that seems to limit and prevent the inhibition of mineralization [2].

Several studies in fish have shown markedly negative effects of skeletal abnormalities on animal welfare, biological performance, product quality, and production costs [7,44]. Use of zebrafish as vivarium animals is currently restricted to hobbyists and laboratory researchers. Nevertheless, skeletal abnormalities, caused by P-deficient diets, must be taken in account because they not only reduce commercial value but also cause bias in the results of studies using this fish (or other fishes) as a model organism. For instance, unaddressed skeletal abnormalities due to dietary deficiencies of phosphorus, calcium, or other ions could affect fish behavior by reducing swimming ability as a result of diminished bone density. Drug and toxicological responses could also be altered due to an imbalance in phosphate homeostasis, which can cause changes in the availability of ATP, which is used for extracellular signaling. Such deficiencies may even interfere in the analysis of muscle hypertrophy by affecting the regular growth of fibers. In other words, neglecting the diet may generate confounding and unreliable results, caused by interference from a new, uncontrolled variable [26]. This can be extrapolated to experiments with other commercial fishes, given the high level of conservation in the functional role of phosphorus in diverse biological pathways.

## 4. Materials and Methods

### 4.1. Experimental Animal Production

The experiment was conducted at the College of Medicine Veterinary and Animal Sciences of the São Paulo State University (FMVZ-UNESP), in the Nutrition of Aquatic Organisms Research Laboratory (AquaNutri, Botucatu, Brazil). All procedures were carried out in accordance with the guidelines for the use of animals, approved by the Association for the Study of Animal Behavior, and were approved by the Sao Paulo State University’s Ethics Committee (109/2014-CEUA, 7 October 2014). The entire trial protocol was carried out in a 90-day period, including the initial phase of animal reproduction and acclimatization (0–30 days after hatching), followed by a second phase of nutritional treatment (31–90 days after hatching). Non-related wild-type (WT) adult zebrafish were randomly crossed to avoid any bias caused by homozygosis. Viable embryos were maintained under optimal water-quality conditions. Four days after hatching, feeding was started with cooked egg yolks and microworms (*Panagrellusredivivus*). After 10 days, larvae were supplemented with saline *Artemia nauplii* until 30 days after hatching. Subsequent treatments are described in detail below.

### 4.2. Nutrition Experiment

Zebrafish (*n* = 450), aged 31 days (after hatching) with average weights of 0.0079 ± 0.0003 g were distributed in a completely randomized experiment design with six treatments and five replications. Each experimental unit was comprised of a single aquarium housing 15 individuals. Thirty aquaria, with volumes of 15 L, were kept at a constant temperature of 28 °C [46]. The aquaria were equipped with water recirculation systems with physical and biological filters to reduce impurities and ammonia levels. The pH, ammonia, and oxygen concentrations were 6.8 ± 0.32, 0.06 ± 0.01 mg L^−1^, and 6.5 ± 0.3 U mg^−1^, respectively, and were measured weekly using a multiparameter YSI 556^®^ (YSI Environmental, Yellow Spring, OH, USA). At the end of the experiment (90 days after hatching), the animals were collected, numbed with clove oil (50 mL L^−1^), sacrificed via benzocaine overdose, and fixed in 5% formaldehyde for 10 days for diaphanization. 

### 4.3. Diets

Semipurified isoproteic and isocaloric diets were formulated based on a reference feed (Table 2) containing 40% digestible protein (DP) and 3500 kcal of digestible energy (DE) [47], which was enhanced with 10 g kg^−1^ of fish flour for palatability. Six distinct diets were prepared to contain total phosphorus contents of 0.35% (T1), 0.65% (T2), 0.95% (T3), 1.25% (T4), 1.55% (T5), and 1.85% (T6). These values were based on the total phosphorus requirement of 0.7–0.9% for carp (*Cyprinus carpio*), which is a closely-related species from the same family (Cyprinidae) [1].

The mineral concentrations of P and Ca were examined at the UNESP Chemistry Laboratory, Botucatu, Sao Paulo, Brazil. Samples were subjected to acid digestion, P quantification was performed according to Markzent [48], and Ca was measured via flame atomic absorption spectrometry using Shimadzu AA-6800 equipment, according to manufacturer protocols (Shimadzu Cookbook Operation Manual: Atomic Absorption Spectrophotometer AA-6800, SHIMADZU-2000). Bromatological diet analysis was carried out in the Laboratory of Bromatology of Breeding and Animal Nutrition Department of FMVZ-UNESP, Botucatu, Sao Paulo, Brazil. Crude protein levels (%) were measured using the Kjedahl method, lipids (%) were measured using Weende’s methodology, and energy (kcal^−1^) was determined by sample combustion in a calorimetric pump (C200, IKA, Staufen, Germany), in accordance with the AOAC-Official Methods of Analysis [49].

### 4.4. Diaphanization

Diaphanization was performed according Pontthoff’s adapted protocol [50]. At the end of the 90-day trial period, two fish per experimental unit (that were fixed in formaldehyde) were washed in distilled water (24 h) and dehydrated in two alcohol solutions (50% and 95%) for 24 h each. For cartilage coloration, fish were immersed for 24 h in an Alcian blue solution (30% Alcian blue diluted in 60% absolute ethanol and 40% acetic acid). Samples were transferred to a borate-saturated solution (5 h), bleaching solution (2 h, 3% H_2_O_2_ in 2% KOH), and a second bleaching solution (7 h, 35% saturated borate, and 65% distilled H_2_O_2_). The samples were then immersed for 24 h in a solution to color the bones (alizarin 2% solution of KOH 2%) and transferred to preservation solution I (24 h, 30% glycerin, and 2% KOH), preservation solution II (24 h, 60% glycerin, and 2% KOH), and a maintenance solution for storage (glycerin and thymol).

### 4.5. Skeletal Deformities

We also evaluated the severity of skeletal anomalies at the end of the trial period in the diaphonized fish. Anomalies were defined using an ascending numerical scale (1 to 6), indicating low to high degrees of severity. This scale was developed by and is routinely used at the AquaNutri Laboratory, FMVZ-UNESP. The intensities of deformities (1 to 6) were counted by visual examination of each individual fish under a stereomicroscope (Stemi 2000, Zeiss). Deformities were classified for severity level according to the standards below described in Table 3. 

### 4.6. Gene Expression

Gene expression analyses were performed in two euthanized animals (90 days after hatching), collected from each experimental unit, with a total of 10 animals analyzed per treatment. Samples were immediately stored in a freezer at −80 °C. For RNA extraction, whole fish samples were individually macerated in liquid nitrogen with a ceramic crucible support and pistil previously autoclaved and decontaminated with RNase AWAY^®^ (Thermo Fisher Scientific, Waltham, MA, USA). 

RNA extraction was performed using the TRIzol method with 500 μL of TRIzol (GIBCO BRL, Waltham, MA, USA) for each sample to disrupt cells and release their contents. The extraction product was visualized on 1% agarose gel and quantified using a NanoDrop instrument (ND-1000 Spectrophotometer, UNESP Botucatu, Brazil). Samples were treated with DNase, and a cDNA synthesis reaction was performed using a mixture containing 0.75 mM of oligodT solution (*n* = 18), 0.15 mM of random oligonucleotides (*n* = 8), 0.75 mM of dNTP, and 11 μL of RNA treated with DNAse from the previous step. The samples were then incubated at 65 °C for 5 min and placed on ice for 1 min. To set the final solution, 0.50 mM of DTT (Dithiothreitol), 40 U of RNase, and 100 U of Super Script III were added. The reaction was then incubated at 50 °C for 1 h and subsequently at 70 °C for 15 min.

Bone mineralization was monitored through quantitative expression analysis (qRT-PCR) of the OC gene transcripts. *ef1α* expression was used as a reference gene for data normalization [51]. Reactions were performed in triplicate on a Real Time ABI 7300 instrument (Applied Biosystems, Foster City, CA, USA) using the SYBR Green PCR Master Mix kit (Applied Biosystems, Foster City, CA, USA) under the following conditions: One cycle at 50 °C for 2 min, another cycle at 94 °C for 10 min, then 40 cycles at 94 °C for 15 s, and 60 °C for 1 min. The obtained dissociation curve was as follows: 95 °C for 15 s, 60 °C for 30 s, and 95 °C for 15 s. Information regarding the primers used for qRT-PCR analyses, nucleotide sequences, and genbank accession numbers is shown in Supplementary Table S1. To calculate the efficiency of the oligonucleotides used, four dilutions of cDNA samples were made: 1:5, 1:25, 1:125, and 1:625. Efficiency (E) was calculated using the formula E = 10 (−1/slope). Relative quantification (R) was determined according to Pfaffl [52]

### 4.7. Statistical Analysis

Skeletal anomaly parameters were subjected to analysis of variance (ANOVA) and the Tukey multiple range test at 5% significance, including the absence of parallelism between the spines, the partial fusion of neural and hemal spines, lack of spine fusion, normal intervertebral spacing, vertebral compression, and relative OC protein gene encoding. Parameters with distinctive values were further checked using a Kruskal-Wallis test at 5% significance. Pearson’s chi-square test was applied for craniofacial anomalies [53]. *p*-Value calculations represent the differences between the groups.

## 5. Conclusions

We concluded that inadequate dietary P levels are critical for the occurrence of several skeletal anomalies in zebrafish. We found that proper zebrafish nutrition requires the ingestion of 1.55% total P. We also demonstrated that OC expression levels were inversely correlated to dietary P levels, which putatively affected both bone mineralization and resorption. We believe that OC expression can be used as a molecular diagnosis marker for monitoring phosphorus deficiency.

## Figures and Tables

**Table 1:** Percentage of fish with vertebral anomalies: Absence of parallelism between neural and hemal spines, partial neural and hemal spine fusion, absence of vertebral body fusion, normal intervertebral spacing, and vertebral compression of zebrafish fed diets with varying phosphorus levels.

Anomalies *	Diets					
	T1	T2	T3	T4	T5	T6
Absence of parallelism between neural and hemal spines	51.5 ^a^	52.0 ^a^	43.0 ^a^	39.0 ^ab^	5.5 ^bc^	0.0 ^c^
Partial neural spine fusion	10.0 ^a^	9.0 ^a^	6.0 ^a^	10.0 ^a^	3.0 ^c^	0.0 ^c^
Partial hemal spine fusion	10.0 ^a^	10.0 ^a^	8.5 ^a^	9.5 ^a^	3.0 ^c^	0.0 ^c^
Absence of vertebral body fusion fusion	35.0 ^ab^	70.0 ^a^	61.5 ^a^	16.0 ^b^	6.0 ^bc^	0.0 ^c^
Normal intervertebral spacing	20.0 ^c^	56.5 ^b^	54.0 ^b^	100.0 ^a^	100.0 ^a^	100.0 ^a^
Vertebral compression	10.0 ^bc^	32.5 ^a^	21.0 ^a^	14.0 ^b^	6.0 ^bc^	0.0 ^c^

* Value in the same row with different superscript letters are significantly different (Kruskal-Wallis test, *p* < 0.0001, *n* = 60).

**Table 2:** Composition of six experimental diets for zebrafish.

Ingredients (%)	Diets					
	T1	T2	T3	T4	T5	T6
Albumin	34.74	34.76	34.79	34.81	34.83	34.85
Poultry Viscera Meal	10.00	10.00	10.00	10.00	10.00	10.00
Gelatin	8.00	8.00	8.00	8.00	8.00	8.00
Starch	38.95	36.01	33.11	30.21	27.31	24.42
Soy oil	2.64	3.51	4.36	5.21	6.07	6.92
Cellulose	5.00	5.00	5.00	5.00	5.00	5.00
Dicalcium phosphate ^a^	0.00	2.05	4.07	6.10	8.12	10.14
BHT ^b^	0.02	0.02	0.02	0.02	0.02	0.02
NaCl	0.10	0.10	0.10	0.10	0.10	0.10
Premix ^c^	0.50	0.50	0.50	0.50	0.50	0.50
Vitamin C ^d^	0.05	0.05	0.05	0.05	0.05	0.05
Total	100.00	100.00	100.00	100.00	100.00	100.00
Chemical Composition *						
Crude Protein %	40.13	40.07	40.00	40.08	40.01	40.10
Crude Lipid %	4.53	5.39	6.24	7.09	7.93	8.78
Total Phosphorus (%)	0.34	0.67	0.93	1.26	1.55	1.81
Ca/P ratio (%)	2.03	2.25	2.32	2.34	2.37	2.38
Energy (kcal^−1^)	3.493	3.494	3.506	3.506	3.508	3.493

^a^ Dihydrate dicalcium phosphate. ^b^ butylated hydroxytoluene. ^c^ Vitamin–mineral premix (composition by kg product): vitamin A = 1.200 UI; vitamin D3 = 200.000 UI; vitamin E = 12.000 mg; vitamin K3 = 2.400 mg; vitamin B1 = 4.800 mg; vitamin B2 = 4.800 mg; vitamin B6 = 4.000 mg; vitamin B12 = 4.800 mg; folic acid = 1.200 mg; calcium pantotenate = 12.000 mg; vitamin C = 48.000 g; biotin = 48 mg; choline = 65.000 mg; niacin = 24.000 mg, iron = 10.000 mg; copper = 600 mg; manganese = 4.000 mg; iodine = 20 mg; cobalt = 2 mg e selenium = 20 mg. ^d^ Vitamin C: Calcium salt, 2-ascorbic acid monophosphate, 35% active principle. * Analyzed composition in the diet.

**Table 3:** Parameters for classifying the intensities of zebrafish deformities in ascending numerical scale (1 to 6).

Deformities	Scale					
	1	2	3	4	5	6
	Number of Vertebrae (V) or Spines (S) Affected					
Scoliosis (V)	1–2	3–4	5–6	7–8	9–10	≥11
Lordosis (V)	1–2	3–4	5–6	7–8	9–10	≥11
Anchylosis (S)	1–2	3–4	5–6	7–8	9–10	≥11
Caudal fin anomaly (V)	1	2	3	4	5	≥6

## Supplementary Materials

Supplementary materials can be found at http://www.mdpi.com/1422-0067/19/2/364/s1.
