# Supplementary material for: Inadequate Dietary Phosphorus Levels Cause Skeletal Anomalies and Alter Osteocalcin Gene Expression in Zebrafish
Source: Int J Mol Sci. 2018 Jan 25;19(2):364. doi: 10.3390/ijms19020364 (PMC5855586; doi:10.3390/ijms19020364)
Supplement: Supplementary file 1 [file ijms-19-00364-s001.pdf]

International Journal of *Molecular Sciences*

MDPI

Article

Supplementary Materials: Inadequate Dietary Phosphorus Levels Cause Skeletal Anomalies and Alter Osteocalcin Gene Expression in Zebrafish

Juliana M. Costa [1,*], Maria M. P. Sartori [2], Nivaldo F. do Nascimento [3], Samir M. Kadri [4], Paulo E. M. Ribolla [5], Danillo Pinhal [6,*] and Luiz E. Pezzato [7]

[1] Department of Genetics, Institute of Biosciences, Sao Paulo State University (UNESP), Botucatu, Sao Paulo 18618-970, Brazil
[2] Department of Crop Science, College of Agricultural Sciences, Sao Paulo State University, Botucatu, Sao Paulo 18610-307, Brazil; mmpsartori@fca.unesp.br
[3] Aquaculture Center, Sao Paulo State University (CAUNESP), Jaboticabal, Sao Paulo 14884-900, Brazil; nivaldotec@yahoo.com.br
[4] Botucatu Biotechnology Institute, Sao Paulo State University (UNESP), Botucatu, Sao Paulo 18618-970, Brazil; samirkbr@yahoo.com.br
[5] Botucatu Biotechnology Institute, Sao Paulo State University (UNESP), Botucatu, Sao Paulo 18618-970, Brazil; pribolla@ibb.unesp.br
[6] Department of Genetics, Institute of Biosciences, Sao Paulo State University (UNESP), Botucatu, Sao Paulo 18618-970, Brazil
[7] College of Veterinary and Animal Science, Sao Paulo State University (UNESP), Botucatu, Sao Paulo 18618-970, Brazil; epezzato@fmvz.unesp
* Correspondence: juh_agro87@yahoo.com.br (J.M.C.); dlpinhal@ibb.unesp.br (D.P.); Tel.: +55-14-99622-4010 (J.M.C.); +55-14-991-213-203 (D.P.)

Table S1. Oligonucleotides sequences used in qPCR and supplementary information.

Gene	Gene bank acess number	Primers sequence 5′-3′	Amplification (pb)	Ta (ºC) [a]	EE (%)[b]
Oc	NM_001083857	CTGCCTGATGACTGTGTGTGTGAGCG<br>GGCGGCATGATTCCAGACG	220	61	87.4
ef1α	NM_131263	ACGCCCTCCTGGCTTTCACCC<br>TGGGACGAAGGCAACACTGGC	217	61	115.0

[a] Ta optimal annealing temperature specific to each oligonucleotide; [b] Measuring the efficiency of the reaction Real Time PCR (calculated trough the standard curve).
